# The Relationship between Inflammation Markers (CRP, IL-6, sCD40L) and Colorectal Cancer Stage, Grade, Size and Location

**DOI:** 10.3390/diagnostics11081382

**Published:** 2021-07-31

**Authors:** Olga Martyna Koper-Lenkiewicz, Violetta Dymicka-Piekarska, Anna Justyna Milewska, Justyna Zińczuk, Joanna Kamińska

**Affiliations:** 1 Department of Clinical Laboratory Diagnostics, Medical University of Bialystok, Waszyngtona 15A, 15-269 Białystok, Poland; justyna.zinczuk@umb.edu.pl (J.Z.); joanna.kaminska@umb.edu.pl (J.K.); 2 Department of Statistics and Medical Informatics, Medical University of Bialystok, Szpitalna 37, 15-295 Białystok, Poland; anna.milewska@umb.edu.pl

**Keywords:** colorectal cancer, biomarker, inflammation, linear regression analysis, prognostic value

## Abstract

The aim of the study was the evaluation whether in primary colorectal cancer (CRC) patients (*n* = 55): age, sex, TNM classification results, WHO grade, tumor location (proximal colon, distal colon, rectum), tumor size, platelet count (PLT), mean platelet volume (MPV), mean platelet component (MCP), levels of carcinoembryonic antigen (CEA), cancer antigen (CA 19-9), as well as soluble lectin adhesion molecules (L-, E-, and P-selectins) may influence circulating inflammatory biomarkers: IL-6, CRP, and sCD40L. We found that CRP concentration evaluation in routine clinical practice may have an advantage as a prognostic biomarker in CRC patients, as this protein the most comprehensively reflects clinicopathological features of the tumor. Univariate linear regression analysis revealed that in CRC patients: (1) with an increase in PLT by 10 × 10^3^/μL, the mean concentration of CRP increases by 3.4%; (2) with an increase in CA 19-9 of 1 U/mL, the mean concentration of CRP increases by 0.7%; (3) with the WHO 2 grade, the mean CRP concentration increases 3.631 times relative to the WHO 1 grade group; (4) with the WHO 3 grade, the mean CRP concentration increases by 4.916 times relative to the WHO 1 grade group; (5) with metastases (T1-4N+M+) the mean CRP concentration increases 4.183 times compared to non-metastatic patients (T1-4N0M0); (6) with a tumor located in the proximal colon, the mean concentration of CRP increases 2.175 times compared to a tumor located in the distal colon; (7) in patients with tumor size > 3 cm, the CRP concentration is about 2 times higher than in patients with tumor size ≤ 3 cm. In the multivariate linear regression model, the variables that influence the mean CRP value in CRC patients included: WHO grade and tumor localization. R^2^ for the created model equals 0.50, which indicates that this model explains 50% of the variance in the dependent variable. In CRC subjects: (1) with the WHO 2 grade, the mean CRP concentration rises 3.924 times relative to the WHO 1 grade; (2) with the WHO 3 grade, the mean CRP concentration increases 4.721 times in relation to the WHO 1 grade; (3) with a tumor located in the rectum, the mean CRP concentration rises 2.139 times compared to a tumor located in the distal colon; (4) with a tumor located in the proximal colon, the mean concentration of CRP increases 1.998 times compared to the tumor located in the distal colon; if other model parameters are fixed.

## 1. Introduction

Colorectal cancer (CRC) is one of the cancers associated with a chronic inflammatory process [1,2]. Cytokines and chemokines produced by cancer attract and activate inflammatory cells and stimulate the release of pro-inflammatory proteins and growth factors by tumor cells.

C-reactive protein (CRP), an acute-phase reactive protein produced by hepatocytes, is generally used for the assessment of inflammation, including cancer-associated inflammation [3,4]. It is upregulated in response to proinflammatory cytokines including IL-1β, IL-6, TNF-α [3,5]. Furthermore, an elevated serum CRP concentration is associated with tumor invasiveness, cancer progression, and a higher recurrence rate in CRC patients [1,6,7].

An important mediator of the inflammatory response is IL-6, and its increased expression may contribute to the development of many inflammatory diseases [8]. It also influences tumor growth directly by interacting with transformed tumor cells and indirectly by activation of stromal cells (tumor milieu) [9]. IL-6, secreted by cancer-associated fibroblasts, activates the JAK/STAT3, PI3K/Akt, and Ras pathways, which are the three major pathways in endothelial cells involved in tumor development. Their stimulation leads to increased proliferation, migration, and invasion of tumor cells, angiogenesis, and increased apoptotic resistance [10]. High IL-6 concentration is a poor prognostic factor, and it is associated with a higher relapse risk and shorter survival time in patients with ovarian cancer, intestinal cancer, bladder cancer, stomach cancer, lung cancer, and pancreas and liver cancer [11,12,13,14,15,16]. It was shown that IL-6 can induce resistance to chemotherapeutics [17]. Recent studies indicate that anti-IL6 therapeutics are able to neutralize IL-6 production and inhibit CRP production in vivo and could be safe and useful in inflammatory diseases [18].

Ligand CD40 (CD40L), and its receptor CD40, are members of the tumor necrosis factor (TNF) superfamily, and are important in the functioning of the immune system [19]. CD40L is a type II transmembrane protein predominantly expressed on activated CD4+ T cells and on platelets membranes in response to platelet activation [20,21]. Although CD40L and its receptor individually do not perform any definite biological function, their interaction results in the generation of an intracellular signal which affects both humoral and cellular immune responses [22]. The interaction of the CD40 ligand with its CD40 receptor stimulates many types of tumors to release pro-inflammatory proteins. After activation of the CD40 receptor, cancer cells release IL-8, IL-6, or TNF, which consequently increases the T-cell-dependent antitumor immune response [23]. The CD40L/CD40 system, in addition to its immunological effect, can limit tumor growth by increasing the apoptosis of tumor cells [24].

In the available literature, there are systematic reviews analyzing the usefulness of circulating CRP and IL-6 evaluation in CRC patients [4,25]. Vainer et al. [25] emphasize that the results concerning the prognostic value of IL-6 concentration in CRC patients were ambiguous, therefore the influence of cancer stage on IL-6 levels should be investigated further to definitely clarify the quality of IL-6 use. Pathak et al. [4] conclude that CRP concentration evaluation may have an advantage in primary and metastatic CRC patients, however available data are not sufficient to allow its use in routine clinical practice. Studies aiming to evaluate sCD40L in CRC patients are very scarce [26,27,28]. In our previous study, we revealed the diagnostic usefulness of sCD40L evaluation in CRC patients, as the area under the receiver-operator curve (AUC) was 0.915 in differentiating cancer from non-cancer patients [28].

Taking into account the fact that clinical usefulness of CRP and IL-6 concentration determination has not been unequivocally confirmed, and considering how little information is available on the determination of sCD40L concentration in patients with colorectal cancer, the aim of the current study was the evaluation of factors that may influence CRP, IL-6, and sCD40L concentrations in CRC patients. Analyzed variables included: age, sex, TNM classification, WHO grade, tumor location, tumor size, concentration of cancer biomarkers (CEA and CA 19-9), and soluble lectin adhesion molecules, as well as platelets morphological parameters.

## 2. Materials and Methods

The study group consisted of 55 primary colorectal cancer (CRC) patients (median age 68 years), who underwent surgical treatment in the 2nd Department of General Surgery and Gastroenterology, Medical University of Bialystok. The CRC patients were retrospectively included based on symptoms, medical history, radiological, colonoscopy, and histological examination results. The colonoscopy examination showed only one neoplastic lesion, and the radiological imaging did not reveal any additional foci or primary tumors in the intestine. This was a de novo lesion in all patients. Blood samples were collected 1 day prior to the surgery. The exclusion criteria encompassed: other neoplasia, received chemo- or radiotherapy before surgery, any symptoms of acute inflammation (redness, swelling, warmth, and pain [29]) as well as inflammatory bowel disease (Crohn's disease, ulcerative colitis) and other diseases affecting the mechanisms regulating intestinal lining integrity (e.g., obesity, type 2 diabetes) [30], a history of familial adenomatous polyposis, or any anti-inflammatory or antiplatelet treatment at least 2 weeks before the blood collection. In the study population, females (53%) were predominant as compared to males (47%). The most common location of the primary lesion was the proximal colon (*n* = 23, 42%); 16 subjects (29%) had the tumor located in the distal colon and 16 (29%) in the rectum. Table 1 presents the clinical and demographical characteristics of the study patients.

According to the TNM classification of the American Joint Committee of Cancer (AJCC) [31], CRC patients were further divided into three subgroups: group A: I/II stage (T1-4N0M0, *n* = 25), group B: III stage (T1-4N1-2M0, *n* = 16), and group C: IV stage (T1-4N1-2M1, *n* = 14). The TNM classification includes the depth of invasion of the bowel wall (T), lymph node metastasis (*n*), and distant metastasis to the liver (M).

The histological malignancy grade of cancerous lesions that existed during surgery was determined with the use of World Health Organization (WHO) criteria [32]: 44 CRC patients had low malignancy grade (G1–2) and 11 CRC patients were with high malignancy grade (G3).

The control group consisted of 27 healthy volunteers, age- and gender-matched individuals, undergoing their periodical check-up at the Medical University Hospital’s Clinic. They had no cancer history and no signs of inflammation/infection or inflammatory bowel disease (Crohn's disease, ulcerative colitis), and the same as the patient’s group, they had not taken any anti-inflammatory and antiplatelet drugs before examination. Also, in the control, the percentage of females (52%) was higher than males (48%).

The study was conducted in accordance with the Helsinki-II-declaration and was approved by the local Bioethics Human Research Committee (Permission No. R-I-002-121-2015). We only recruited colorectal cancer patients and healthy volunteers who gave their written informed consent.

### 2.1. IL-6, sCD40L, sL-Selectin, sE-Selectin and sP-Selectin Concentrations Evaluation

Plasma samples (3.2% sodium citrate as an anticoagulant, 2.7 mL, S-Monovette, SARSTEDT) were collected after centrifugation for 15 min at 1000× *g* within 30 min of collection. Plasma obtained was immediately separated into aliquots and stored at −75 °C, until the assay time. Prior to the assay, the plasma samples were gradually mixed using a vortex (tecnoKartel). Plasma concentrations of IL-6, sCD40L, sL-selectin, sE-selectin, and sP-selectin were quantified by specific ELISA kits (R&D Systems, Abingdon, UK) following the manufacturer’s instructions. The manufacturer of the assay kit referred to the intra-assay coefficient of variation (CV%) as: 4.2% at IL-6 mean concentration of 16.8 pg/mL ± 0.7 pg/mL; 5.4% at sCD40L mean concentration of 2.64 ng/mL ± 0.14 pg/mL; 5.0% at sL-selectin mean concentration of 210 ng/mL ± 10.5 ng/mL; 6.9% at sE-selectin mean concentration of 1.6 ng/mL ± 0.11 ng/mL. 7.9% at sP-selectin mean concentration of 1.27 ng/mL ± 0.11 ng/mL.

### 2.2. PLT, MPV and MCP Evaluation

Blood samples (2.6 mL) were collected into test tubes containing EDTA-K3 (S-Monovette, SARSTEDT). PLT, MPV, and MCP were automatically determined in the same sample on the hematological analyzer XN-1000 (Sysmex, Kobe, Japan).

### 2.3. CRP, CEA and CA 19-9 Evaluation

Blood samples (2.7 mL) were collected into test tubes without anticoagulant (S-Monovette, SARSTEDT). Within 1 hour of the venipuncture blood was centrifuged for 15 min. at 1000× *g* to obtain serum. CRP concentration was determined by means of the immunoturbidimetry method on the Cobas Integra^®^ 400 plus analyzer (Roche Diagnostics, Basel, Switzerland). CEA and CA 19-9 were analyzed on the Architect ci 8200 biochemical analyzer (Abbott Diagnostics, Abbott Park, IL, USA).

### 2.4. Statistical Analysis

The results were subjected to statistical analysis using the STATISTICA 12.0 PL software (StatSoft Inc., Tulsa, OK, USA) and STATA 12.1 (StataCorp LP, College Station, TX, USA). The Shapiro–Wilk test was used to assess if the features are consistent with normal distribution. To compare results between two groups, the U Mann–Whitney test was applied. The Kruskal–Wallis test was applied for the comparison of a larger number of groups. In the group of CRC patients, we performed a univariate as well as multiple linear regression analysis, to indicate factors that may influence circulating CRP, IL-6, and sCD40-L concentrations. Tested factors included: age, sex, platelet count (PLT), mean platelet volume (MPV), mean platelet component (MCP), levels of carcinoembryonic antigen (CEA), cancer antigen (CA 19-9), soluble lectin adhesion molecules (L-, E-, and P-selectins) as well as TNM classification results, WHO grade, tumor location (proximal colon, distal colon, rectum), and tumor size. After the final model was obtained, residuals were calculated. Then, to assess the normality of the residuals, a normal probability plot was performed. A residual-vs-predictor plot was performed to assess the residual homoscedasticity. Initially, statistically significant variables were included in the univariate linear regression analysis, and then the presented multivariate model was obtained by backward stepwise regression.

A level of *p* < 0.05 was considered statistically significant. If not stated otherwise, results are presented as the median with 25th and 75th percentiles.

## 3. Results

### 3.1. Inflammatory Response Biomarkers (CRP, IL-6, sCD40-L) and Cancer Biomarkers (CEA, CA 19-9) in CRC Patients versus Control Group

**Figure 1:**
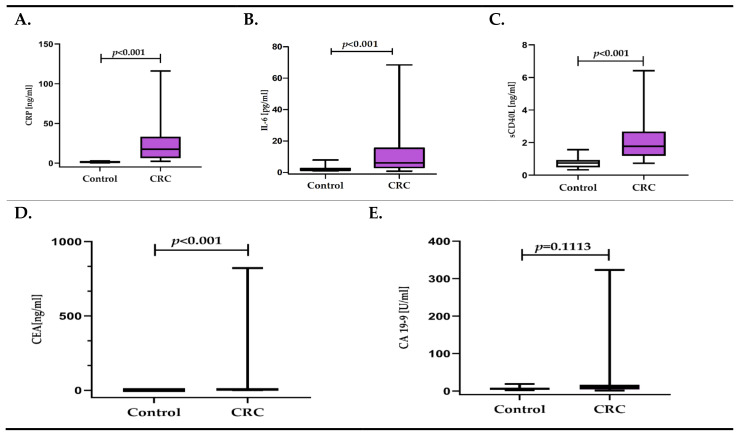
(**A**–**E**). Concentration of inflammatory response biomarkers (CRP, IL-6, sCD40L) and cancer biomarkers (CEA, CA 19-9) in colorectal cancer patients (CRC), in relation to the control group.

The concentrations of all inflammatory response biomarkers tested were significantly higher in the CRC group compared to the control group: CRP concentration was 16.8-fold higher, IL-6 concentration was 2.9-fold, while the sCD40L concentration was 2.4-fold higher. From cancer biomarkers, only CEA concentration significantly differentiated CRC patients from healthy individuals (Figure 1A–E, Table S1).

### 3.2. Inflammatory Response Biomarkers (CRP, IL-6, sCD40-L) and Cancer Biomarkers (CEA, CA 19-9) Depending on TNM Classification

**Figure 2:**
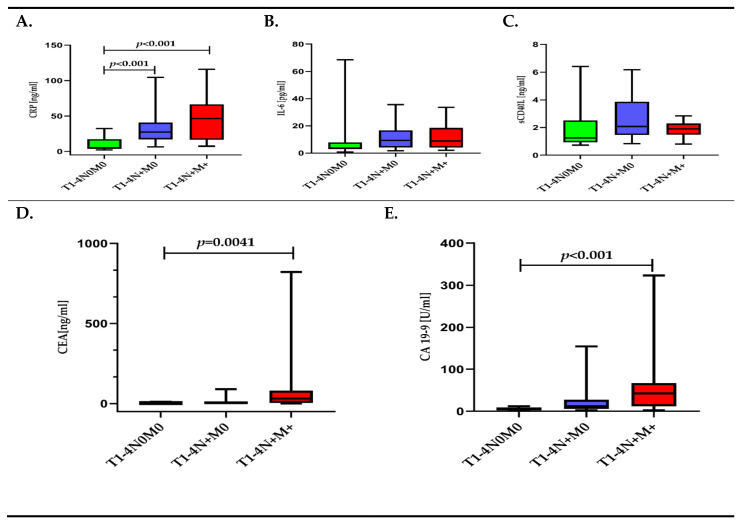
(**A**–**E**). Concentration of inflammatory response biomarkers (CRP, IL-6, sCD40L) and cancer biomarkers (CEA, CA 19-9) in colorectal cancer patients (CRC) depending on the TNM classification.

Analysis of the CRP, IL-6, and sCD40-L concentrations, depending on the TNM classification, showed statistical differences only for CRP. Patients with the tumor limited to the intestinal wall (T1-4N0M0) had a 5.2-fold lower CRP concentration compared to patients with lymph node metastases (T1-4N+M0) and an almost 9-fold lower CRP concentration compared to patients with lymph node and distant metastases to the liver (T1-4N+M+). The IL-6, as well as the sCD40L concentrations, reached the highest values in the group of patients only with lymph node metastases. Both CEA and CA 19-9 were differerent depending on the TNM classification (Figure 2A–E, Table S1).

### 3.3. Inflammatory Response Biomarkers (CRP, IL-6, sCD40-L) and Cancer Biomarkers (CEA, CA 19-9) Depending on the Presence of Metastasis

**Figure 3:**
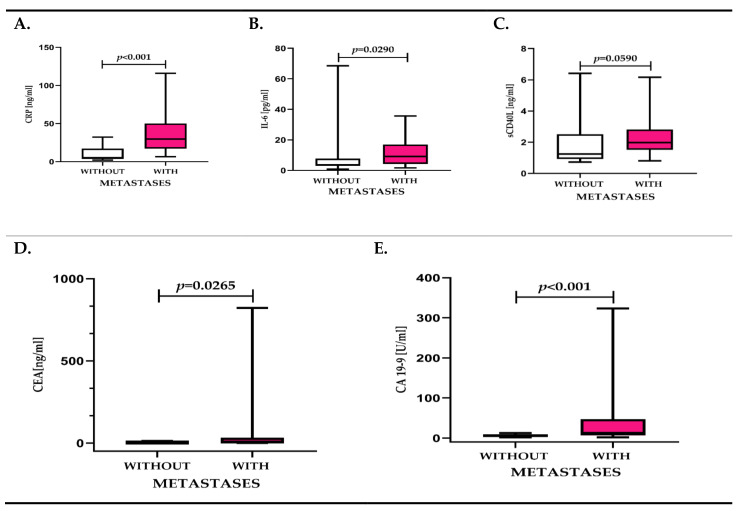
(**A**–**E**). Concentration of inflammatory response biomarkers (CRP, IL-6, sCD40L) and cancer biomarkers (CEA, CA 19-9) in colorectal cancer patients (CRC) depending on the presence of metastases.

Analyzing the tested molecules, depending on the presence of metastasis, we found statistical differences for CRP and IL-6, but not for sCD40L. IL-6 and CRP concentrations in patients with lymph node and/or distant metastases to the liver were 2.7-fold and 5.7-fold higher, respectively, compared to patients without metastases. The sCD40L concentration in metastatic patients was also higher compared to individuals without metastases, but it was not statistically significant. Also, CEA and CA 19-9 were significantly higher in patients with lymph node and/or distant metastases to the liver compared to patients without metastases (Figure 3A–E, Table S1).

### 3.4. Inflammatory Response Biomarkers (CRP, IL-6, sCD40-L) and Cancer Biomarkers (CEA, CA 19-9) Depending on WHO Grade

**Figure 4:**
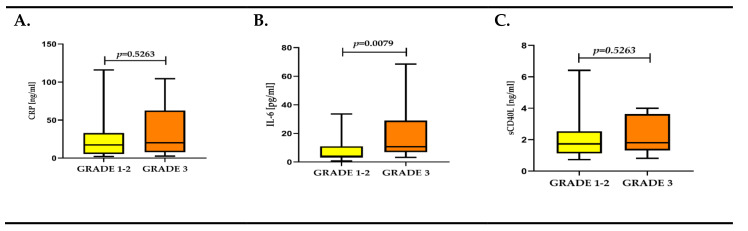

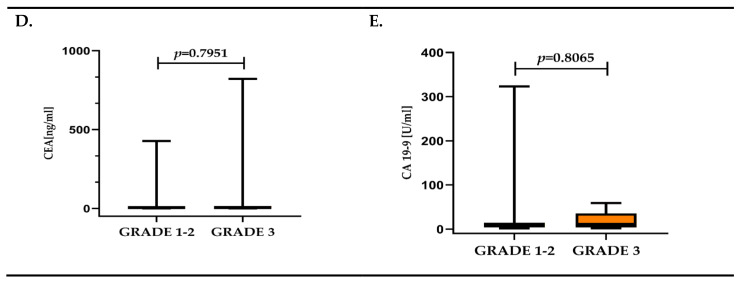
(**A**–**E**). The concentration of inflammatory response biomarkers (CRP, IL-6, sCD40L) and cancer biomarkers (CEA, CA 19-9) in colorectal cancer patients (CRC) depending on the grade.

Analysis of CRP, IL-6, and sCD40L concentrations, depending on the WHO grade, indicated that only the IL-6 concentration differentiated these patients, as its levels were 2.6-fold higher in WHO 3 grade patients compared to WHO 1–2 grades patients. The remaining molecules, CRP and sCD40L, were also higher in WHO 3 grade patients compared to WHO 1–2 grade patients, but the obtained differences were not significant. Neither CEA nor CA 19-9 significantly differentiated patients, depending on the WHO grade (Figure 4A–E, Table S1).

### 3.5. Univariate and Multivariate Linear Regression Analysis for CRP

In CRC patients: (1) with an increase in PLT by 10 × 10^3^/μL, the mean concentration of CRP increases 1.034 times (increases by 3.4%); (2) with an increase in CA 19-9 of 1 U/mL, the mean concentration of CRP increases 1.007 times (increases by 0.7%); (3) with the WHO 2 grade, the mean CRP concentration increases 3.631 times relative to the WHO 1 grade group; (4) with the WHO 3 grade, the mean CRP concentration increases by 4.916 times relative to the WHO 1 grade group; (5) with metastases (T1-4N+M+) the mean CRP concentration increases 4.183 times compared to non-metastatic patients (T1-4N0M0); (6) with a tumor located in the proximal colon, the mean concentration of CRP increases 2.175 times compared to a tumor located in the distal colon; (7) in patients with tumor size > 3 cm, the CRP concentration is about 2 times higher than in patients with tumor size ≤ 3 cm (Table 2).

In the multivariate linear regression model, the variables that influenced the mean CRP value in CRC patients included: WHO grade and tumor localization. R2 for the created model equals 0.50, which indicates that this model explains 50% of the variance in the dependent variable. In CRC subjects: (1) with the WHO 2 grade, the mean CRP concentration rises 3.924 times relative to the WHO 1 grade; (2) with the WHO 3 grade, the mean CRP concentration increases 4.721 times in relation to the WHO 1 grade; (3) with a tumor located in the rectum, the mean CRP concentration rises 2.139 times compared to a tumor located in the distal colon; (4) with a tumor located in the proximal colon, the mean concentration of CRP increases 1.998 times compared to the tumor located in the distal colon; if other model parameters are fixed (Table 2).

### 3.6. Univariate and Multivariate Linear Regression Analysis for IL-6

In the CRC patients: (1) with an increase of PLT by 10 × 10^3^/μL, the mean concentration of IL-6 increases 1.036 times (increases by 3.6%); (2) with an MPC increase of 1 g/dL, the average concentration of IL-6 increases 1.30 times (increases by 30%), (3) with sE-selectin increase of 1 ng/mL, the average concentration of IL-6 increases 1.022 times (increases by 2%); (4) with a CRP increase of 1 ng/mL, the average concentration of IL-6 increases 1.012 times (increases by 1.2%); (5) in the group of metastatic patients (T1-4N+M+), the mean IL-6 concentration increases 1.73 times compared to the non-metastatic patients (T1-4N0M0) (increases by 73%) (Table 3).

In the multivariate linear regression model, the variables that influenced the mean IL-6 value in the CRC patients included: PLT and MPC. Adjusted R square (R2) for the created model equals 0.26, which indicates that this model explains 26% of the variance in the dependent variable. In CRC patients: (1) with an increase in PLT by 10 × 10^3^/μL, the mean concentration of IL-6 increases 1.04 times (increases by 4%); (2) with an MPC increase of 1 g/dL, the average concentration of IL-6 increases 1.28 times (increases by 28%), if other model parameters are fixed (Table 3).

### 3.7. Univariate and Multivariate Linear Regression Analysis for sCD40L

In the CRC patients: (1) with an increase of PLT by 10 × 10^3^/μL, the mean sCD40L concentration increases 1.023 times (increases by 2.3%); (2) with an MPC increase of 1 g/dL, the average concentration of sCD40L increases 1.15 times (increases by 15%); (3) with the WHO 2 grade, the mean sCD40L concentration increases 1.48 times relative to the WHO 1 grade (increases by 48%) (Table 4).

In the multivariate linear regression model, the variables that influence the mean sCD40L value in the CRC patients included: PLT and MPC. R2 for the created model equals 0.33, which indicates that this model explains 33% of the variance in the dependent variable. In the CRC patients: (1) with an increase of PLT by 10 × 10^3^/μL, the mean concentration of sCD40L increases 1.024 times (increases by 2.4%); (2) with an MPC increase of 1 g/dL, the average concentration of sCD40L increases 1.14 times (increases by 14%), if other model parameters are fixed (Table 4).

### 3.8. Diagnostic Utility of Inflammatory Response Biomarkers (CRP, IL-6, sCD40L) and Cancer Biomarkers (CEA, CA 19-9)

All inflammatory response biomarkers tested (CRP, IL-6, sCD40L) showed higher area under the ROC curves (AUCs) compared to CEA when discriminating CRC patients from the control group. Also their Youden Indexes (YIs) were higher as compared to CEA. However the highest AUC, YI, diagnostic sensitivity, specificity and accuracy had CRP (Table 5). When discriminating CRC patients without metastases from patients with lymph node and distant metastases, the highest AUC and the YI was also found for CRP (Table 5).

## 4. Discussion

Worldwide, CRC is the fourth and third leading cause of cancer-related death in men and women, respectively [25]. The influence of chronic inflammation on the development of CRC is confirmed by numerous studies [1,5,6,10]. They indicate the presence of inflammatory cells in the tumor microenvironment, which, as a result of activation, release a number of growth factors, cytokines, and chemokines. These pro-inflammatory factors have an impact on tumor development and formation, accelerate the inflammation around the tumor tissues, and increase the production of subsequent inflammatory proteins. According to Pathak et al. [4], the evaluation of inflammatory biomarkers in CRC patients may suggest prognosis and advise the choice of treatment. Therefore, due to increasing treatment options, there is a need to better explore inflammatory response biomarkers, such as IL-6, CRP, and sCD40L, which have the potential to be useful in CRC prognosis [4,25,28].

In respect to the above-mentioned issues, we focused on the following factors in the current study: age, sex, TNM staging, WHO grade, tumor location (proximal colon, distal colon, rectum), tumor size, the levels of carcinoembryonic antigen (CEA), cancer antigen (CA 19-9), soluble lectin adhesion molecules (L-, E-, and P-selectins), platelet count (PLT), mean platelet volume (MPV), and mean platelet component (MCP), that may influence circulating IL-6, sCD40L, and CRP concentrations in CRC patients. Factors, which we chose to be potentially associated with IL-6, sCD40L, and CRP levels, were analyzed by means of linear regression models.

In the present cohort of CRC patients, we found that factors influencing IL-6 concentration included PLT, MPC, sE-selectin, CRP, and TNM stage. IL-6 plays an important role in modulating the immune response in cancer. The chronic inflammatory process, in which IL-6 is released from activated inflammatory cells and CEA-dependent activated Küpfer cells, is responsible for the elevated concentration of this cytokine [33]. Other studies have shown that besides cancer cells, tumor-associated macrophages (TAMs) and cancer-associated fibroblasts (CAFs) also produce and release IL-6 [34,35].

Nagasaki et al. [10] demonstrated that fibroblasts accompanying tumor cells are able to synthesize larger amounts of this cytokine, and suggested that cancer cells stimulate CAFs to produce IL-6. According to Liu et al. [35], high IL-6 expression by CAFs could induce strong immunosuppression in the hepatocellular carcinoma (HCC) microenvironment by recruiting immunosuppressive cells. It was revealed that persistent increased IL-6 concentration, after tumor resection, has an unfavorable value in predicting the presence of distant metastases. And even by some, it is considered to be an independent negative predictor of survival in cancer patients.

In our current study, the IL-6 concentration was 2.9-fold higher in patients with CRC than in the control group. Our previous study presented that IL-6 concentration significantly decreased 12 days after colorectal cancer resection, as compared to values obtained 3 days after surgery [36]. As IL-6 is one of the main inflammatory markers, these results confirm the existence of inflammation in the course of CRC. Univariate linear regression analysis also showed that in the group of metastatic CRC patients (T1-4N+M+), the mean IL-6 concentration increases 1.73 times compared to non-metastatic patients (T1-4N0M0). These results may indicate a direct relationship between poor cancer prognosis and IL-6 concentration. Moreover, IL-6 may be considered as a predictive risk biomarker of CRC recurrence due to its relationship with lymph node metastasis, which is still recognized as the most gainful prognostic factor [37]. It was revealed that persistent increased IL-6 concentration, after tumor resection, had an unfavorable value in predicting the presence of distant metastases [38]. And even by some, it is considered to be an independent negative predictor of survival of cancer patients [39].

Multivariate linear regression analysis showed that in CRC patients with a PLT increase by 10 × 10^3^/μL and with an MPC increase by 1 g/dL, the mean IL-6 concentration increased by 4% and 28%, respectively. It may indicate that platelets, despite their role in thrombotic processes, are directly involved in inflammation accompanying tumorigenesis [40]. This hypothesis is further supported by our remaining findings, as both CRP and sCD40L levels were also increasing with the PLT rise. To better understand this phenomenon, it should be noted that tumor cells, via presenting various glycoproteins, interact with platelets leading to their activation, which results in the release of proinflammatory mediators such as PAF, TXA2, sP-selectin, sCD40L, serotonin, histamine, PF4, and others [40]. Research concerning the CD40 ligand demonstrated its commitment to the development of the inflammatory process in various diseases, including cardiovascular diseases and atherosclerosis [12], as well as inflammatory bowel diseases such as colitis ulcerosa or Crohn’s disease [41].

Recent reports also indicated that CD40L is involved in the development of cancer [42]. It is suggested that the primary source of membrane CD40L in the course of malignancies are lymphocytes and platelets, while the soluble plasma ligand (sCD40L) is primarily derived from active platelets [42]. The results of our study may confirm this theory, as the linear regression analysis presented a relationship between PLT and sCD40.

In the current study, the sCD40L concentration was 2.4-fold higher in CRC patients than in healthy subjects. In the available literature, there is little information regarding the role of sCD40L in colorectal cancer [26,27,28], and it is further believed that the role of the CD40 ligand is not the same in different cancers. Li et al. [43] showed significantly higher concentrations of sCD40L in patients with gastric cancer compared with healthy subjects, which, moreover, was associated with the progression of cancer. Ning et al. [44] found that in the development of this tumor, the CD40 ligand supported angiogenesis by activating the VEGFR receptor and increased VEGF production. Huang et al. [45] suggested that the CD40/CD40L pathway is involved in the suppression of the immune response, while a study by Büning et al. [46] indicated the antitumor activity of the ligand. The authors suggested that an increased expression of CD40L on the surface of stimulated T lymphocytes may be relevant for the generation of anti-tumor immune responses via the activation of antigen-presenting cells (APC), or by direct interaction with the CD40 receptor on tumor cells. As shown by Georgopoulos et al. [47], there is an increased expression of the CD40 receptor on the surface of transformed bowel cancer cells, whose presence is not reported on healthy intestinal cells. The authors contemplated that the activation of the receptor by the CD40 ligand results in the inhibition of cell growth or cell death. Increased expression of adhesion proteins on the surface of activated endothelial cells promotes the expression of CD40L by platelets, which may influence cancer progression by facilitating the formation of metastases [28,48].

Increased expression of adhesion proteins stimulated by sCD40L may affect cancer progression by facilitating metastasis, as we have shown in our current research. In our studied CRC group, the sCD40L concentration increased depending on the WHO grade and was also higher in metastatic patients compared to non-metastatic patients. Moreover, linear regression analysis showed that in WHO 2 grade CRC patients, the mean sCD40L concentration increased by 48% relative to the WHO 1 grade CRC patients, which strongly supports the role of sCD40L in colorectal cancer progression and may indicate this protein as a potential biomarker of colorectal cancer advancement.

We also found that the CRP concentration in CRC patients was 16.8-fold higher compared to non-cancer individuals. McMillan et al. [49] showed that elevated preoperative CRP concentration was related to worse patient prognostication after CRC resection. Their findings may be partially explained by our findings, indicating that with a CRP increase of 1 ng/mL the average concentration of IL-6 increases by 1.2%. The role of IL-6 in the progression of CRC was proven, however, is not fully understood [34,50,51]. It is suggested that it stimulates the growth of tumor cells in vivo via para- and autocrine activation.

Among factors influencing CRP concentration in CRC patients, we found: PLT, CA 19-9, WHO grade, TNM stage, and tumor location. Thus, our findings indicate CRP as a molecule most comprehensively reflecting CRC advancement and progression. CRP also has another advantage: it is cheap and routinely analyzed in laboratories [4].

Pathak et al. [4] performed a systematic review regarding the usefulness of CRP in CRC prognostication. Based on the literature analysis, the authors concluded that CRP has the potential to be helpful in the prognosis of primary and metastatic CRC patients, however, the clinical usefulness of this protein in routine clinical practice has to be validated [4]. In this context, our analysis gives a new insight into the use of CRP concentration evaluation in CRC patients. Linear regression analysis showed that among all proteins analyzed, only CRP was related to the WHO grade, TNM stage, and, likewise, tumor location. Moreover, the adjusted R square (R2) for the created multiple linear regression analysis indicated that the obtained model explains 50% of the variance in CRP concentration in our cohort of CRC patients. R2 presents how much variance can be explained by a model [52].

We found, that in the CRC WHO 2 grade and WHO 3 grade patients the mean CRP concentration rises 3.924 times and 4.721 times in relation to the WHO 1 grade subjects, respectively. This could be a direct relationship of CRP concentration with the CRC progression. Interestingly, multivariate analysis performed by Yamamoto et al. [53] revealed that the combination of pre- and postoperative CRP levels was an independent prognostic indicator of the CRC patients’ survival.

We also found that with a tumor located in the rectum, the mean CRP concentration rises 2.13 times compared to a tumor located in the distal colon, whilst with a tumor located in the proximal colon, the mean concentration of CRP increases 1.998 times compared to the tumor located in the distal colon. Our findings are in contrast to Song et al. [54], who did not present the association between neither CRP nor IL-6 levels and CRC location. In their studies, only the macrophage inhibitory cytokine-1 (MIC-1) concentrations gradually increased from adenomas located in the rectum, distal colon, and up to the proximal colon [54].

### Study Limitations

The lack of a full prospective power analysis could be considered as one of the study limitations. However, such analysis is usually carried out in big cooperative studies including cohorts. Our purpose was more modest—we only aimed to evaluate which factors may influence circulating CRP, IL-6, and sCD40L concentrations in CRC patients. Moreover, to definitively evaluate the prognostic and predictive value of CRP and IL-6, their concentrations should be analyzed with relation to the overall and cancer-specific survival rate [55]. We do not have such data, and therefore we were not able to conduct this kind of analysis, which could also be considered as a study limitation. We are also aware that that current issue requires further research on a larger group of patients as a small number of participants was another limitation of our study.

## 5. Conclusions

Searching for circulating biomarkers useful not only in the CRC diagnosis but are also helpful in the assessment of cancer progression, and forecast is still of great interest [56]. The evaluation of inflammatory biomarkers: IL-6, CRP, and sCD40L, may be helpful in CRC diagnosis, indicating disease advancement, prognosis, and even advising the choice of treatment. Therefore, due to increasing treatment options, there is a need to better explore IL-6, CRP, and sCD40L, which have the potential to be useful in CRC management.

We found that in CRC patients, factors influencing the IL-6 concentration included PLT, MPC, sE-selectin, CRP, and TNM stage. The sCD40L concentrations were influenced by PLT, MPC, and WHO grade. Among factors influencing the CRP concentration, we found PLT, CA 19-9, WHO grade, TNM stage, tumor size, and location. Thus, our findings indicate that CRP concentration evaluation in routine clinical practice may have an advantage as a prognostic biomarker in CRC patients, as this protein most comprehensively reflects the clinicopathological features of the tumor. However, to make a solid conclusion on the CRP prognostic value in CRC patients, future studies should be conducted with regard to the CRP relationship to overall and cancer-specific survival rates.

## Figures and Tables

**Table 1:** Colorectal cancer patient demographical and clinical data. Age is presented as the median with 25th and 75th percentiles.

Variable	Group A	Group B	Group C
	T_1-4_N_0_M_0_	T_1-4_N_+_M_0_	T_1-4_N_+_M_+_
	*n* = 25	*n* = 16	*n* = 14
Age	71 (60–77)	60 (54–70)	69 (63–73)
Sex			
male/female	11/14	7/9	8/6
Grading			
G1/G2/G3	2/19/4	2/10/4	0/11/3
Depth of tumor invasion			
T1-T2	7	1	1
T3-T4	18	15	13
Lymph node			
N0 (absent)	25	0	0
N1+2 (present)	0	16	14
Distant metastasis			
M0 (absent)	25	16	0
M1 (present)	0	0	14
Tumor location			
Proximal colon	9	7	7
Distal colon	7	6	3
Rectum	9	3	4
Tumor size			
≤3 cm	14	1	4
>3 cm	11	15	10

**Table 2:** Univariate and multivariate linear regression analysis results for CRP in colorectal cancer patients.

**No.**	**Covariate**	**Univariate Linear Regression Analysis**		
		**β**	**e^β^ (95%CI)**	***p*-Value**
1	PLT [x10^3^/µL]	0.003	1.003 (1.000–1.006)	0.030
2	CA 19-9 [U/mL]	0.006	1.007 (1.001–1.012)	0.019
	WHO 1	base group		
3	WHO 2	1.290	3.633 (2.144–6.151)	<0.001
4	WHO 3	1.592	4.916 (2.838–8.516)	<0.001
5	Metastases (T_1-4_N_+_M_+_)	1.431	4.183 (2.679–6.531)	<0.001
	Distal colon tumor location	base group		
6	Rectum tumor location	0.601	1.824 (0.864–3.851)	0.112
7	Proximal colon tumor location	0.777	2.175 (1.093–4.327)	0.028
8	Tumor size	0.713	2.040 (1.130–3.685)	0.019
**No.**	**Covariate**	**Multivariate Linear Regression Analysis**		
		**β**	**e^β^ (95%CI)**	***p*-Value**
	WHO 1	base group		
1	WHO 2	1.367	3.924 (2.386–6.456)	<0.001
2	WHO 3	1.552	4.721 (2.819–7.906)	<0.001
	Distal colon tumor location	base group		
3	Rectum tumor location	0.761	2.139 (1.235–3.706)	0.008
4	Proximal colon tumor location	0.692	1.998 (1.209–3.303)	0.008

**Table 3:** Univariate and multivariate linear regression analysis results for IL-6 in colorectal cancer patients.

**No.**	**Covariate**	**Univariate Linear Regression Analysis**		
		**β**	**e^β^ (95%CI)**	***p*-Value**
1	PLT [x10^3^/µL]	0.004	1.004 (1.001–1.006)	0.008
2	MPC [g/dL]	0.268	1.307 (1.050–1.626)	0.018
3	sE-selectin [ng/mL]	0.022	1.022 (1.005–1.039)	0.011
4	CRP [ng/mL]	0.012	1.012 (1.003–1.022)	0.007
5	Metastases (T_1-4_N_+_M_+_)	0.546	1.726 (1.054–2.828)	0.031
**No.**	**Covariate**	**Multivariate Linear Regression Analysis**		
		**β**	**e^β^ (95%CI)**	***p*-Value**
1	PLT [x10^3^/µL]	0.004	1.004 (1.001–1.007)	0.005
2	MPC [g/dL]	0.250	1.284 (1.051–1.568)	0.016

**Table 4:** Univariate and multivariate linear regression analysis results for sCD40L in colorectal cancer patients.

**No.**	**Covariate**	**Univariate Linear Regression Analysis**		
		**β**	**e^β^ (95%CI)**	***p*-Value**
1	PLT [x10^3^/µL]	0.002	1.002 (1.001–1.004)	0.002
2	MPC [g/dL]	0.143	1.153 (1.031–1.290)	0.014
	WHO 1	base group		
3	WHO 2	0.395	1.484 (1.062–2.074)	0.022
4	WHO 3	0.147	1.158 (0.817–1.641)	0.403
**No.**	**Covariate**	**Multivariate Linear Regression Analysis**		
		**β**	**e^β^ (95%CI)**	***p*-Value**
1	PLT [x10^3^/µL]	0.002	1.002 (1.001–1.004)	0.001
2	MPC [g/dL]	0.132	1.141 (1.034–1.259)	0.010

**Table 5:** Diagnostic utility of inflammatory response biomarkers (CRP, IL-6, and sCD40L) and cancer biomarkers (CEA and CA 19-9) for discriminating CRC patients from healthy controls (A), and for discriminating CRC patients without metastases from patients with lymph node and distant metastases (B).

	AUC ± SE	*p*-Value	Youden Index	Cut-Off	Sensitivity	Specificity	Diagnostic Accuracy
A: Discriminating CRC Patients from Healthy Controls							
CRP	0.996 ± 0.004	<0.001 *	0.94	2.43	98%	96%	98%
IL-6	0.864 ± 0.042	<0.001 *	0.69	2.88	84%	85%	84%
sCD40L	0.920 ± 0.029	<0.001 *	0.65	1.20	73%	93%	79%
CEA	0.771 ± 0.054	<0.001 *	0.56	2.50	67%	89%	75%
B: Discriminating CRC Patients without Metastases from Patients with Lymph Node and Distant Metastases							
CRP	0.885 ± 0.043	<0.001 *	0.61	19.52	73%	88%	80%
IL-6	0.671 ± 0.075	0.0229 *	0.41	4.31	77%	64%	71%
CEA	0.686 ± 0.079	0.0179 *	0.42	9.44	46%	96%	69%
CA 19-9	0.808 ± 0.064	<0.001 *	0.55	11.60	65%	90%	76%

* *p* < 0.05, statistically significant.

## Data Availability

The datasets generated and/or analyzed during the current study are not publicly available, but are available from the corresponding author (O.M.K.-L.) upon request.

## Supplementary Materials

The following are available online at https://www.mdpi.com/article/10.3390/diagnostics11081382/s1. Table S1. Concentration of inflammatory response biomarkers (CRP, IL-6, sCD40L) and cancer biomarkers (CEA, CA 19-9) in colorectal cancer patients (CRC) in relation to the control group and depending on clinicopathological characteristics of tumor. Q1—25th percentile, Q3—75th percentile.
