# Supplementary material for: The Relationship between Inflammation Markers (CRP, IL-6, sCD40L) and Colorectal Cancer Stage, Grade, Size and Location
Source: Diagnostics (Basel). 2021 Jul 31;11(8):1382. doi: 10.3390/diagnostics11081382 (PMC8393680; doi:10.3390/diagnostics11081382)
Supplement: Supplementary file 1 [file diagnostics-11-01382-s001.zip › diagnostics-1247165-supplementary.pdf]

Table S1. Concentration of inflammatory response biomarkers (CRP, IL-6, sCD40L) and cancer biomarkers (CEA, CA 19-9) in colorectal cancer patients (CRC) in relation to the control group and depending on clinicopathological characteristics of tumor. Q1 – 25th percentile, Q3 – 75th percentile.

		**CRP [ng/ml]**		**IL-6 [pg/ml]**		**sCD40L [ng/ml]**		**CEA [ng/ml]**		**CA 19-9 [U/ml]**	
		**Median (Q1-Q3)**	***p*-value**	**Median (Q1-Q3)**	***p*-value**	**Median (Q1-Q3)**	***p*-value**	**Median (Q1-Q3)**	***p*-value**	**Median (Q1-Q3)**	***p*-value**
**Group tested**	CRC	17.35 (6.19-33.20)	<0.001*	6.11 (3.12-15.42)	<0.001*	1.77 (1.18-2.68)	<0.001*	3.11 (1.52-10.02)	<0.001*	9.95 (4.50-15.00)	0.1113
	Control	1.03 (0.56-1.88)		2.13 (1.37-2.56)		0.75 (0.48-0.93)		0.99 (0.85-2.06)		6.18 (3.75-8.15)	
**TNM classification**	T1-4N0M0 (Group A)	5.21 (3.85-17.02) [@,#]	<0.001*	3.43 (3.11-7.72)	0.0944	1.25 (0.94-2.48)	0.1045	2.59 (1.20-3.84) [&]	0.0052*	4.84 (2.41-8.74) [$]	0.006*
	T1-4N+M0 (Group B)	27.35 (17.81-39.01)		9.27 (4.37-16.39)		2.08 (1.50-3.84)		3.08 (0.91-9.44)		11.60 (6.10-27.61)	
	T1-4N+M+ (Group C)	46.44 (17.35-64.94)		8.83 (4.31-17.46)		1.91 (1.58-2.23)		31.50 (3.61-80.90)		42.60 (11.90-67.30)	
**Metastases**	without metastases	5.21 (3.85-17.02)	<0.001*	3.43 (3.11-7.72)	0.0290*	1.25 (0.94-2.48)	0.0590	2.59 (1.20-3.84)	0.0265*	4.84 (2.41-8.74)	<0.001*
	with metastases	29.6 (17.35-49.30)		9.16 (4.31-16.78)		1.98 (1.54-2.81)		4.41 (2.50-31.50)		12.89 (6.69-43.58)	
**Grade**	Grade 1-2	17.19 (5.70-32.65)	0.5263	4.15 (3.11-10.99)	0.0079*	1.73 (1.15-2.52)	0.5263	3.08 (1.41-10.60)	0.7951	9.80 (4.50-13.78)	0.8065
	Grade 3	20.13 (7.78-62.47)		10.71 (6.94-29.0)		1.81 (1.32-3.63)		3.28 (2.52-9.44)		11.60 (4.88-27.61)	

*p <0.05, statistically significant

@ – statistically significant when compared to Group B in post hoc test, p<0.001

# – statistically significant when compared to Group C in post hoc test, p<0.001

& – statistically significant when compared to Group C in post hoc test, p=0.0041

$ – statistically significant when compared to Group C in post hoc test, p<0.001
